# Study on the Influence of Laser Power on the Heat–Flow Multi-Field Coupling of Laser Cladding Incoloy 926 on Stainless Steel Surface

**DOI:** 10.3390/ma17194769

**Published:** 2024-09-28

**Authors:** Linjie Li, Quanwei Cui, Jianxing Zhou, Zhicheng Lu, Haoran Sun, Hong Jiang, Wanli Guo, An Wu

**Affiliations:** 1 School of Mechanical Engineering, Xinjiang University, Urumqi 830047, China; llj15517476003@163.com (L.L.); xju_zhjx@xju.edu.cn (J.Z.); m18827518205@163.com (Z.L.); inevitablehran@163.com (H.S.); 13546217134@163.com (W.G.); 2 Goldwind Science and Technology Co., Ltd., Beijing 100010, China; wuan@goldwind.com

**Keywords:** laser cladding, laser power, temperature field, velocity flow field, microstructure

## Abstract

In order to explore the influence of laser power on the evolution of molten pool and convective heat transfer of laser cladding Incoloy 926 on stainless steel surface, a three-dimensional thermal fluid multi-field coupled laser cladding numerical model was established in this paper. The variation of latent heat during solid-liquid phase transformation was treated by apparent heat capacity method. The change in the gas–liquid interface was tracked using the mesh growth method in real time. The instantaneous evolution of temperature field and velocity flow field of laser cladding Incoloy 926 on a stainless steel surface under different laser power was discussed. The solidification characteristic parameters of the cladding layer were calculated based on the temperature-time variation curves at different nodes. The mechanism of the impact of laser power on the microstructure of the cladding layer was revealed. The experiment of laser cladding Incoloy 926 on 316L surface was carried out under different laser power. Combined with the numerical simulation results, the effects of laser power on the geometrical morphology, microstructure and element distribution of the cladding layer were compared and analyzed. The results show that with the increase in laser power, the peak temperature and flow velocity of the molten pool surface both increase significantly. The thermal influence of the molten pool center on the edge is enhanced. The temperature gradient, solidification rate, and cooling rate increased gradually. The microstructure parameters (G/R) are relatively small when the laser power is 1000 W. In the experimental range, the dilution rate and wetting angle of the cladding layer both increase with the increase in laser power. When the laser power is 1000 W, the alloying elements of the cladding layer are more evenly distributed and the microstructure is finer. The experimental results are in good agreement with the simulation results.

## 1. Introduction

Laser cladding, as an advanced and environmentally friendly surface modification technology, can utilize a high energy density laser beam to rapidly melt the substrate and the cladding powder and realize the metallurgical bond between the two [1]. It can obtain a composite coating with high bonding strength, fine organization and good comprehensive performance [2], and then, it can realize high quality and efficient surface strengthening and performance repair [3]. This technology effectively solves the problems of simple processing, simple structure and poor molding quality in the traditional composite coating preparation method [4]. Stainless steel has good high-temperature mechanical properties and is relatively inexpensive to manufacture, which is why it is widely used in the aerospace, petrochemical, construction, and medical fields [5,6]. However, due to the harsh working environment and high mechanical loads, stainless steel is often subject to wear, rupture, pitting and other failures during long-term service in various fields [7,8,9]. The service life of stainless steel workpieces is greatly limited. Therefore, the use of laser cladding technology to strengthen the stainless steel workpiece can improve its reliability and service life. This can create great economic value and has become a hot topic of concern for scholars at home and abroad [10,11]. In the laser cladding process, the laser power directly determines the thermal energy input of the laser beam to the cladding layer [12]. It is a key factor affecting the organization and mechanical properties of the cladding [13]. M.H. Nie et al. [14] analyzed the formability, structural evolution, mechanical properties and interfacial bond strength of 17-4PH stainless steel cladding layers at different laser powers. It was found that the aspect ratio and dilution of the cladding layer increased when the laser power increased. The strength at the interface increased and the plasticity decreased. However, too-high laser power caused the microstructure to become rough. Shaoxiang Qian et al. [15] prepared three different power levels of nickel-based alloy cladding coatings on 316L stainless steel. It was found that the microstructure transformed to more equiaxed dendrites and columnar dendrites with the increase of laser power. E. W. A. Figueredo et al. [16] evaluated the effect of laser power in the laser melting process of 316L austenitic and 431 martensitic stainless steels. In the cladding process of two different materials, they found that the laser power was the main factor affecting the melting zone. The higher the power value, the greater the dilution of the two deposits and the better the wettability. Rivero S H E L et al. [17] deposited Hastelloy c-276 alloy (TM) coatings on the surface of 304L stainless steel and GGG40 ductile iron using different laser powers. They found that as the laser power increased, the dilution rate of the two different types of cladding increased and the microstructure of both became finer. Their hardness and wear resistance were also significantly enhanced.

However, laser cladding is a transient, fast-heating and fast-cooling process that involves complex thermodynamic behavior and multi-physical field coupling [13]. The real-time evolution of the melt pool during the cladding process cannot be accurately described by experiment alone [12]. The emergence of simulation software has facilitated the application of the finite element method (FEM) in the characterization process of laser cladding [18,19]. Yanle Li et al. [20] used ANSYS to establish a thermodynamic coupling model to analyze the influence law of different heat treatment conditions on the temperature field and stress field of laser cladding 316L.They found that preheating significantly increased the temperature of the melt pool. The annealing treatment had the best effect on the improvement of laser melting cladding residual stresses. Gaosong Li et al. [21] and Feng Weifeng et al. [22] developed a two-dimensional axisymmetric numerical model of transient laser polishing. The temperature distribution, velocity magnitude, and melt pool evolution of the 316L surface polishing model were simulated. It is found that the surface curvature of the melt pool determines the dominant law of thermal capillary force on the flow direction of the molten material. When the surface curvature of the bath is large, the molten material flows from the peaks to the troughs under the dominance of capillary forces. If the surface of the bath is smooth, the molten material will flow towards the edge of the bath under the dominance of thermal capillary force. Bilel Si Smail et al. [23] developed a repair numerical chain for pitting damaged components and investigated the numerical optimization of ellipsoid pre-machining during laser repair of 316L parts. It was found that the numerical optimization greatly reduced the amount of repair. The microstructure of the repaired area was effectively refined, and the mechanical properties were significantly improved.

**Figure 1:**
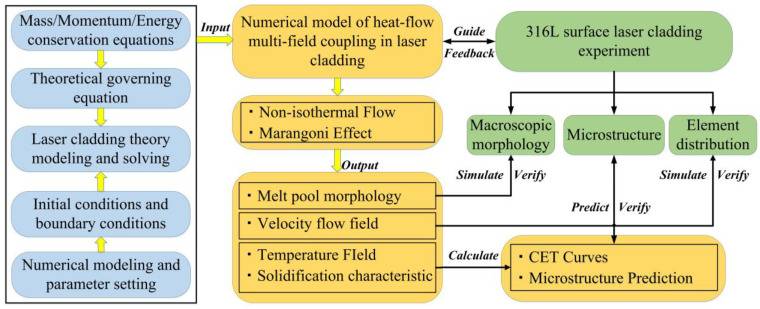
The research ideas of this study.

In summary, numerical simulation analysis is an effective method to study the molten pool evolution and convective heat transfer during laser cladding of stainless steel surface, which has been iteratively studied by many scholars. However, most of the current numerical models focus on thermal-force coupling. The effects of powder flow and molten pool evolution are often ignored when the boundary conditions are set. There is a lack of regularity in the study of temperature-driven flow on the heat–flow interaction in the laser cladding process. At the same time, the research on the relationship between the microstructure and solidification characteristics of the cladding layer, the relationship between the distribution of elements and the evolution of the flow field both needs to be improved. In this paper, the addition of powder, the non-isothermal flow of molten pool and the dynamic evolution of solidification structure were fully considered. A three-dimensional numerical model of laser cladding with multi-field coupling of heat and flow was established by COMSOL simulation software(Version 5.6). The model included the laser-powder interaction and the dynamic growth of the cladding layer. The variation of latent heat during the solid–liquid phase transformation was treated by apparent heat capacity method. The gas-liquid interface was captured dynamically by mesh growth method. According to the simulation results, the variation laws of temperature field, flow field, and molten pool morphology during laser cladding under different laser power were studied. The solidification characteristics were obtained by studying the thermal behavior of the molten pool, and the effect of laser power on the solidification structure of the cladding was analyzed. Laser cladding experiments were carried out on a 316L stainless steel surface under different power conditions. Combined with the morphology, solidification characteristics and flow field evolution of the molten pool obtained by numerical simulation, the geometric morphology, microstructure and element distribution of the cladding layer under different power were studied. In this study, the effect of laser power on the multi-field coupling of heat flow of laser cladding Incoloy 926 on stainless steel surface cladding was discussed. It also provided an important reference for predicting the geometric characteristics and microstructure evolution of cladding layer by numerical simulation method. The basic research ideas of this paper are shown in Figure 1.

## 2. **Materials and Experimental** Methods

For the experiment, 316L stainless steel with specifications of 21 × 10 × 5 mm^3^ was selected as the substrate. Its surface was sandblasted to enhance the adhesion of the powder on the substrate and to prevent laser reflection damage to the laser head. Incoloy 926 alloy powder was selected as the experimental cladding powder. The powder needs to be dried in a drying oven before the experiment and set aside. The chemical composition of the experimental materials is shown in Table 1.

**Figure 2:**
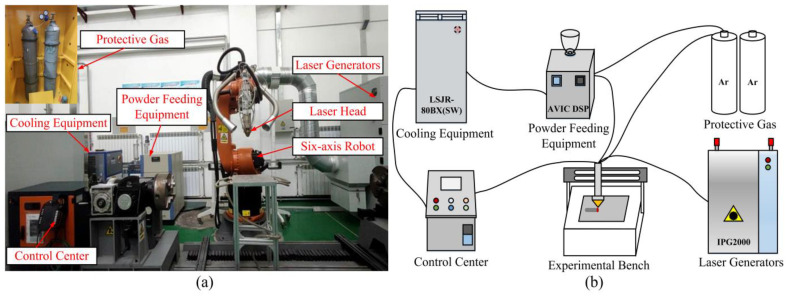
Construction of the experimental platform for laser cladding: (**a**) experimental equipment; (**b**) schematic diagram.

The laser cladding experimental platform is mainly composed of fiber laser generator (IPG2000, Oxford, MA, USA), six-axis robot (KUKA, Augsburg, Bavaria, Germany), powder feeder (AVIC DSP, Beijing, China),water cooler for laser (LSJR-80BX (SW), Zhuhai, China) and protective inert gas cabinet, as shown in Figure 2. The protective gas is argon, which has a purity greater than or equal to 99.99%. After the experiments were completed, the fused specimens were cut into metallographic specimens of size 8 × 8 × 8 mm^3^ using an EDM cutter. The metallographic specimens were repeatedly polished with 600 mesh, 800 mesh, 1000 mesh, 1200 mesh, 1500 mesh, 1800 mesh and 2000 mesh sandpaper. And use the metallographic grinding and polishing machine to polish the sample until there are no scratches. The polished specimens were corroded for 3–4 min using an etching solution with a ratio of hydrochloric acid: nitric acid = 3:1. Then the surface was scrubbed with anhydrous ethanol. Finally, the specimens were dried with a hair dryer. The metallographic microstructure of the specimens was initially observed using an ordinary optical microscope (OM) to confirm the corrosion effect. The microstructure and element distribution of the cladding layer was observed using scanning electron microscopy (SEM) and energy spectroscopy (EDS). The grain size of the cladding microstructure was measured using the Nano Measurer software (Version 1.2), and then data analysis was performed.

## 3. Laser Cladding Theory Modeling and Solving

The melt pool evolution during laser cladding is a highly transient behavior with many influencing factors. Considering the computational cost and the accuracy of the research directions, the numerical model is established based on the following simplifying assumptions [24,25]:

(1) The laser energy is assumed to obey a Gaussian distribution, and the heat input is stabilized, which acts directly on the surface of the molten pool.
(2) The powder stream concentration is assumed to obey a Gaussian distribution, and it melts immediately after falling into the melt pool.
(3) The metal melt is assumed to be an incompressible Newtonian fluid, and the flow inside the molten pool is laminar.
(4) The thermophysical parameters of the substrate and the powder are assumed to be temperature dependent and to exhibit isotropy.
(5) The effect of carrier and protective gases on the melt pool is assumed to be neglected.

### 3.1. General Control Equations for Laser Cladding

The equations for the conservation of mass, momentum, and energy during laser cladding are given by Equations (1)–(3) [26]:

(1) $$\frac{\partial \rho }{\partial t}+\nabla \cdot (\rho u)=0$$

(2) $$\rho [\frac{\partial u}{\partial t}+(u\cdot \nabla u)]=\nabla \cdot [-PI+\mu (\nabla u+{\left(\nabla u\right)}^{T})]-\frac{2\mu }{3}(\nabla \cdot u)I]-\psi_{0}\frac{{\left(1-f_{l}（T）\right)}^{2}}{f_{l}（T{）}^{3}+\varphi }u$$

(3) $$\rho C_{p}\frac{\partial T}{\partial t}+\rho C_{p}u\cdot \nabla T=\nabla \cdot (k\nabla T)-\frac{\partial H}{\partial t}-\rho u\cdot \nabla H$$

where *ρ* is the density, u is the velocity vector, *μ* is the hydrodynamic viscosity, *P* is the pressure, *I* is the unit matrix, *F* is the volumetric force acting on the fluid (viscous resistance and drag), *Ψ*_0_ is a constant determined by the morphology of the porous medium; *φ* is a very small parameter avoiding the denominator to be zero, *Cp* is the specific heat capacity, *T* is the temperature, *k* is the heat transfer coefficient, and H is the latent enthalpy of the molten metal.

The latent enthalpy, or potential enthalpy, is numerically equal to the amount of heat required to change the metal from a solid to a liquid during isobaric melting and can be expressed as:

(4) $$△H=L\cdot f_{l}(T)$$

where *L* is the potential heat during the phase transition and *f_l_*(*T*) is the liquid mass fraction, which can be expressed by the following equation:

(5) $$f（T{）}_{l}=\left\{\begin{array}{ccc} 1 & ， & T>T_{l}\\ \frac{T-T_{s}}{T_{l}-T_{s}} & ， & T_{s}\leq T\leq T_{l}\\ 0 & ， & T<T_{s} \end{array}\right.$$

where *T* is the temperature and l and s represent the liquid and solid phase lines, respectively.

In the interval (*T_s_*, *T_l_*), there exists a “mushy zone” of mixed material properties. The latent heat is treated using the apparent heat capacity method to accurately capture the latent heat of phase change in the mushy zone. This ensures a smooth transition of the solid–liquid form. In other words, the latent heat is included in the heat capacity as an additional term [27]:

(6) $$C_{p}=\frac{（1-f_{l}(T)）\rho_{s}C_{p,s}+f_{l}(T)\rho_{l}C_{p,l}}{\rho }+L\frac{df_{l}(T)}{dT}$$

### 3.2. Initial and Boundary Conditions for Numerical Solution

The initial temperature of the object before the start of laser cladding can be expressed as:

(7) $${\left.T(x,y,z,t)\right|}_{t=0}=T_{amb}$$

where *T_amb_* is the ambient temperature (293.15 K).

At the surface of a molten pool, heat transfer between gas and liquid occurs at all times [28]. Therefore, heat flow boundary conditions need to be defined to determine the way heat transfer occurs inside and outside the melt pool, which can be expressed as:

(8) $$-n\cdot q=Q+h(T_{amb}-T)+\varepsilon \sigma (T_{amb}^{4}-T^{4})$$

where the three terms on the right side of the equation are the laser Gaussian heat source heat flux, convective loss heat flux, and external radiation heat flux respectively. *h* is the heat transfer coefficient, *ε* is the surface emissivity, and σ is the Stefan–Boltzmann constant. All planes except the gas–liquid interface are adiabatic boundaries with a heat flux of 0.

To ensure that the laser irradiation penetrates into the interior of the molten pool, the laser energy input is modeled as a Gaussian body heat source, assuming that the distribution function can be expressed as [29]:

(9) $$Q_{L}=\frac{6aP_{L}}{\pi^{\frac{3}{2}}\cdot {R_{L}}^{2}\cdot h}\exp[-3\cdot (\frac{{\left(x-v_{L}\cdot t\right)}^{2}+y^{2}}{{R_{L}}^{2}}+\frac{z^{2}}{h^{2}})]$$

where *P_L_* is the laser power, a is the laser absorption rate, *R_L_* is the radius of the laser beam spot, h is the effective cladding depth and *v_L_* is the scanning speed. The Gaussian heat source model can adjust the size of the effective heat-affected zone of laser cladding by changing the range of values of *x*, *y* and *h*, so as to make corresponding changes for different cladding characteristics.

During the laser cladding process, the molten pool interface evolves dynamically and instantaneously. In the arbitrary Lagrangian–Eulerian (ALE) method, the motion of the grid nodes is flexible. It can accurately track the free interface as well as effectively characterize the sharp changes in the motion of fluid plasmas. Therefore, the deformation geometry method based on the arbitrary Lagrangian–Eulerian (ALE) method is utilized to describe the surface morphology evolution process of the melt pool, which can be expressed as follows [30,31]:

(10) $$V_{L/G}=u\cdot n+V_{mesh}\cdot n$$

where *u* is the local flow velocity in the melt pool section, *n* is the normal vector of the gas–liquid interface and *V_mesh_* is the velocity of the interface movement induced by the metal powder into the molten pool, which can be expressed as [27]:

(11) $$V_{\mathrm{mesh}}=\frac{2\eta_{p}v_{p}}{\rho_{p}\pi r_{p}^{2}}\exp[-\frac{2({\left(x-v_{L}\cdot t\right)}^{2}+y^{2})}{r_{p}^{2}}]z$$

where *η_p_* is the capture rate of powder by the melt pool, *v_p_* is the powder feeding rate, and *z* is the unit vector in the z direction.

### 3.3. Numerical Modeling and Parameter Settings

**Figure 3:**
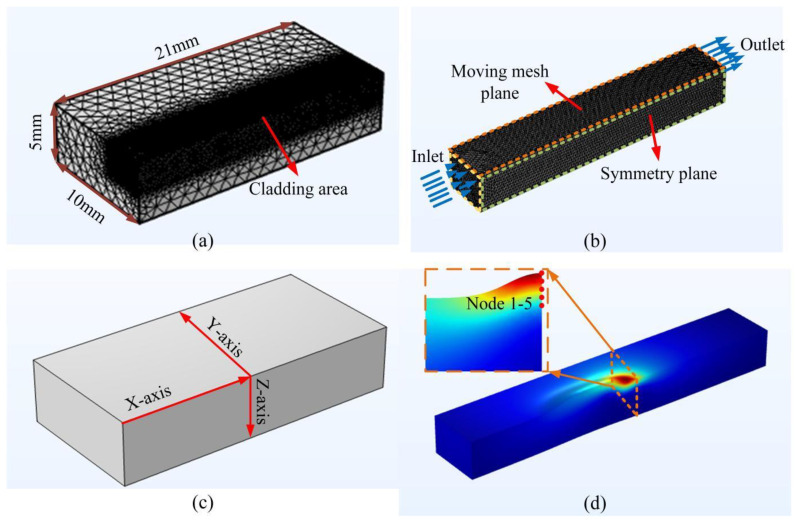
Finite element model of laser cladding: (**a**) meshing; (**b**) cladding area; (**c**) path selection; (**d**) node selection.

The COMSOL simulation software is used to establish a finite element model for numerical simulation of laser cladding heat–fluid multi-field coupling. Set 316L stainless steel and Incoloy 926 alloy as the base and cladding powders in the model respectively. In order to minimize the computational cost, only half workpieces are considered on the basis of ensuring convergence and computational accuracy. The dimensions are 21 × 10 × 5 mm^3^. Free tetrahedral meshing is used to customize the degree of encryption of the meshing. The mesh contains 330,345 domain cells, 15,130 boundary elements and 595 edge cells. The minimum grid size of the melting area was 5 μm and the maximum grid size was 224 μm, as shown in Figure 3. The process parameters during laser cladding are shown in Table 2.

**Figure 4:**
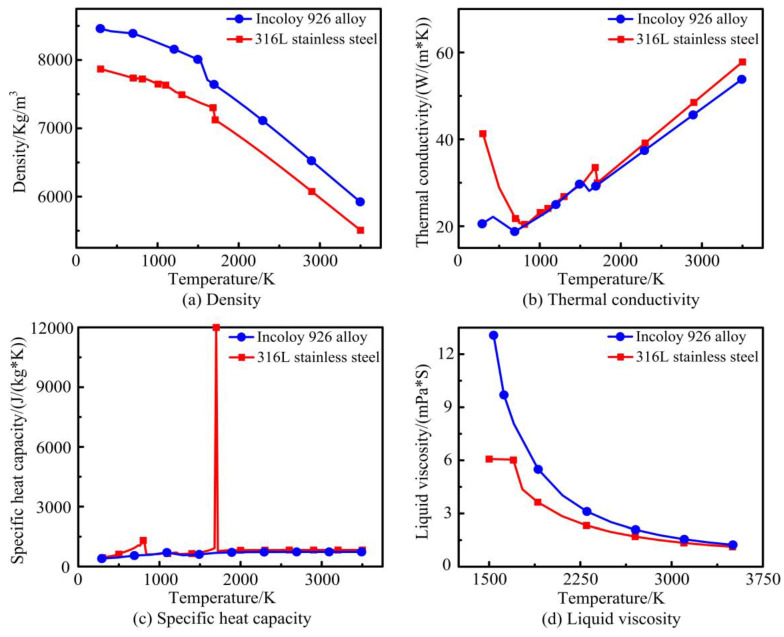
Material thermophysical property parameters: (**a**) density; (**b**) thermal conductivity; (**c**) specific heat capacity; (**d**) liquid viscosity.

During the actual processing of laser cladding, the thermophysical parameters of both the substrate and the powder vary with temperature [30]. Therefore, real-time changes in the thermophysical parameters of metal materials have an important impact on the accuracy of the simulation results. According to the elemental composition of the materials, the thermophysical parameters of 316L stainless steel and Incoloy 926 alloy were obtained by JMatPro software (Version 7.0), as shown in Figure 4. The substrate and powder form a molten pool under laser irradiation. The intense internal Marangoni flow causes a thorough mixing of the alloying elements in the molten substrate and powder. The thermophysical parameters of the molten pool also change and can be expressed as [32]:

(12) $$\phi_{m}=\lambda \phi_{t}+(1-\lambda )\phi_{p}$$

where φ can be replaced by density, thermal conductivity, specific heat capacity, and liquid viscosity and *λ* is determined by the ratio of the cross-sectional areas of the two. The symbols “*m,t,p*”refer to the melt pool, the substrate, and the powder, respectively.

## 4. Analysis of Numerical Simulation Results

### 4.1. Analysis of Temperature Field

**Figure 5:**
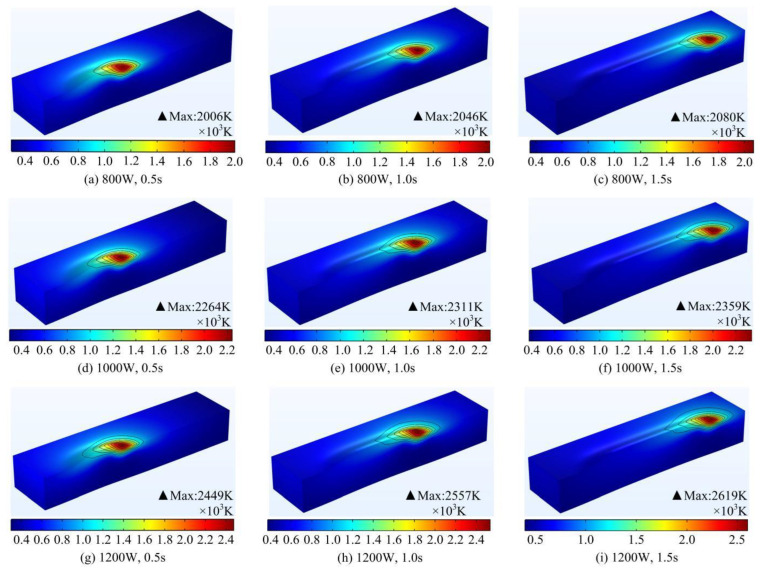
Simulation results of temperature field of molten pool under different laser powers.

In the laser cladding process, the reasonable regulation of the cladding temperature is an important measure to ensure the quality of cladding and process stability. It has an important influence on the formation of molten pool, phase change heat transfer and solidification and crystallization [19,32]. The variation rule of the temperature field of the molten pool in the process of melting and nudging was calculated in the case of different laser powers respectively, as shown in Figure 5. The surface of the melt pool has an elliptical shape. This is due to the fact that when the laser spot is close to a node, the substrate and powder heat up rapidly and reach their melting points and then melt smoothly. While the cladding area has a slower cooling rate due to heat transfer from the melt pool. As a result, the melt pool exhibits a “trailing” behavior. From the temperature field at a fixed power, it can be seen that the peak temperature of the molten pool increases and then stabilizes as the laser beam melting time progresses. The heat-affected zone also reaches its maximum value. Additionally, because of heat transfer and convection, the heat of the melt pool is constantly exchanged with the external environment. This phenomenon is exacerbated by the violent Marangoni flow inside the melt pool, which in turn leads to fluctuations in the peak temperature of the melt pool surface. The temperature field at a fixed moment in time shows that the peak temperature of the molten pool increases with increasing laser power. As the laser power increases, the laser beam outputs more thermal energy, which acts on the molten pool and causes the surface of the molten pool to reach a higher peak temperature.

**Figure 6:**
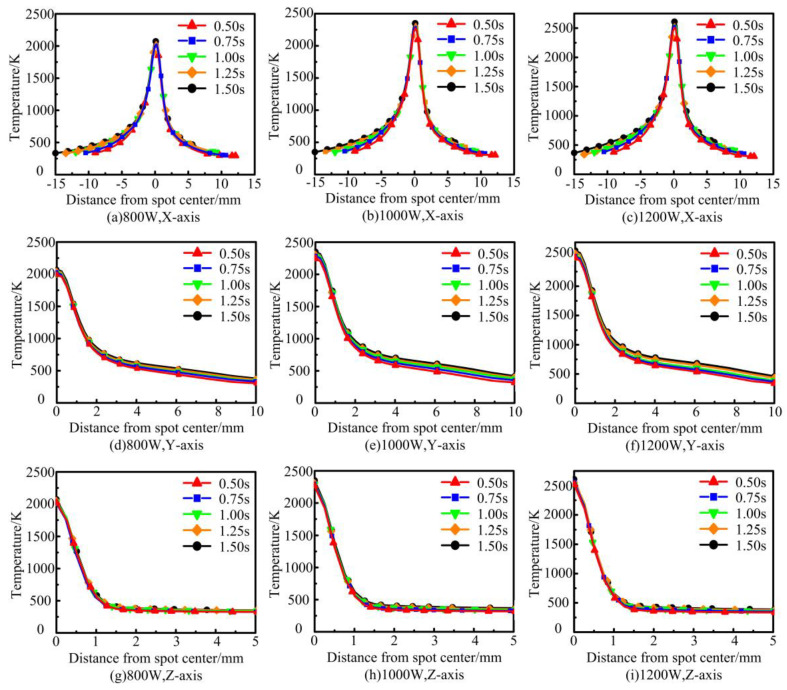
Simulation results of the axial temperature curve of molten pool under different laser powers.

To further explore the law of the instantaneous change of the temperature in the region around the molten pool at different laser powers, three axial (X, Y, Z) temperature acquisition lines were set up on the substrate as shown in Figure 3c. The thermal influence effect law of the molten pool center on the axial region under different laser powers was obtained, as shown in Figure 6. The instantaneous temperature in the X-axis direction shows a clear Gaussian distribution. This is related to the fact that the laser heat energy density is Gaussian-distributed. Using the center of the melt pool as a boundary, the rate of temperature change in the coated area is lower than that in the area to be coated. This is due to the fact that the coated area has a certain temperature base compared to the area to be coated at room temperature. The heat transfer from the laser spot reduces its cooling rate. Along the Y-axis of the melt pool, there is a decreasing trend in temperature away from the spot center. The rate of temperature decrease increases and then decreases with time. Along the Z-axis of the melt pool depth, the temperature away from the center of the spot decreases steeply and finally tends to be the same. As the laser power increases, the peak temperature at the center of the melt pool rises continuously. The closer to the center of the melt pool, the higher the temperature and the faster the rate of temperature change. In addition, as the laser power increases, the depth at which the temperature in the Z-axis direction converges to room temperature increases slightly. This indicates an increase in the depth of the melt pool.

### 4.2. Analysis of the Velocity Flow Field

**Figure 7:**
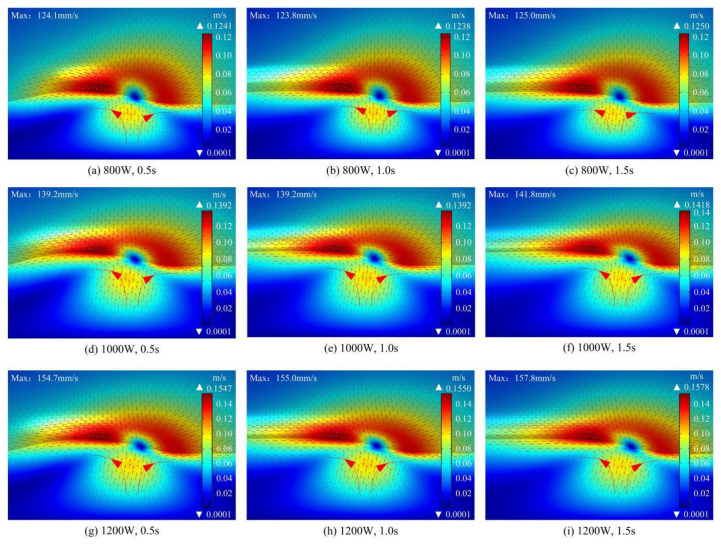
Simulation results of velocity flow field of molten pool under different laser powers.

Melt pool flow in the laser cladding process can promote heat transfer, uniform element distribution, and grain refinement and directly control the melt pool morphology [33]. It has a vital impact on the forming quality and properties of the cladding layer [34]. The distribution of the velocity flow field in the melt pool at different laser powers is shown in Figure 7, with the red arrow indicating the direction of melt flow. As the laser scanning time progresses, the substrate and powder rapidly melt and form a melt pool under the irradiation of the high energy laser beam. The molten metal in the melt pool is stabilized as a Marangoni flow. It flows clockwise at the front end of the laser spot and counterclockwise at the back end of the spot at different times, which is symmetrically distributed. This is due to the large temperature difference between the center and the surroundings of the melt pool, which causes the melt pool to flow from the inside out. At the same time, the flow on the surface of the molten pool induces uneven tensions and generates gradients, which in turn promotes this effect to occur drastically. As the cladding time progresses, both the surface temperature of the melt pool and the flow rate become progressively larger. The Marangoni effect thus becomes more intense. Comparing the velocity flow fields at different powers, laser beam with a higher power generates a greater heat input, causing significant changes in the temperature gradient and surface tension gradient inside the melt pool. This promotes the convection of the melt, which in turn enhances the wetting and mobility within the melt pool. Therefore, when the laser power increases, the velocity of the flow in the melt pool gradually increases.

**Figure 8:**
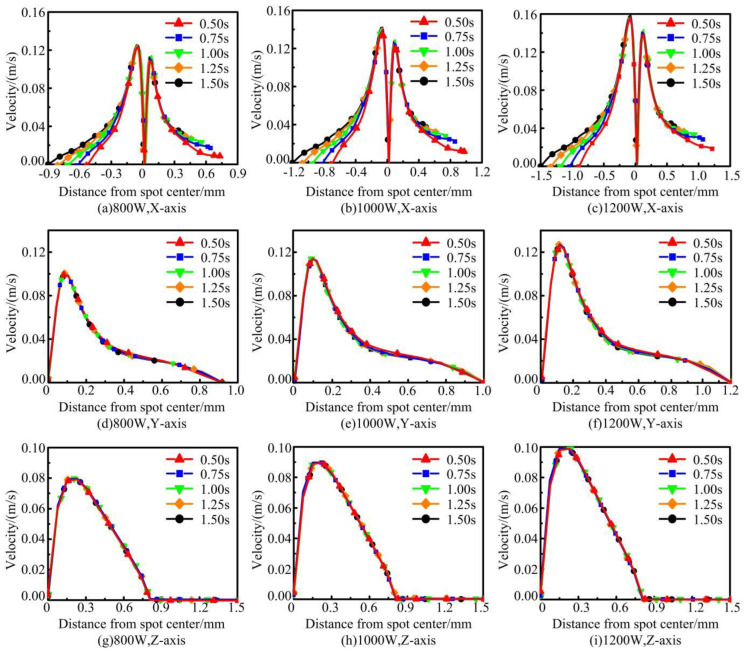
Simulation results of axial velocity curve of molten pool under different laser powers.

The variation curves of the velocity of the molten pool in each axial direction are shown in Figure 8. The molten metal at the center of the melt pool has the lowest flow velocity. While the melt in the region around the melt pool has a larger flow velocity under the Marangoni effect. In the X-axis direction, the molten pool flow rate shows “bimodal”, which is related to the change rule of the temperature curve on both sides of the molten pool. In general, there is a negative correlation between temperature and tension [35]. The tension at the center of the melt pool is the smallest because it has the largest peak temperature. Similarly, the tensions around the melt pool are relatively large because of their small peak temperatures. The molten metal flows from locations of low tension to locations of high tension [36]. In the Y-axis and Z-axis directions, the flow velocity first increases gradually to the peak and then decreases with an increase in distance from the center of the melt pool. With the increase of laser power, both the temperature gradient and tension gradient of the melt pool increase significantly, which makes the melt flow velocity increase consequently. In addition, the intense Marangoni flow induced by high power caused the rate of change of the melt flow rate to increase.

### 4.3. Analysis of Melt Pool Morphology

**Figure 9:**
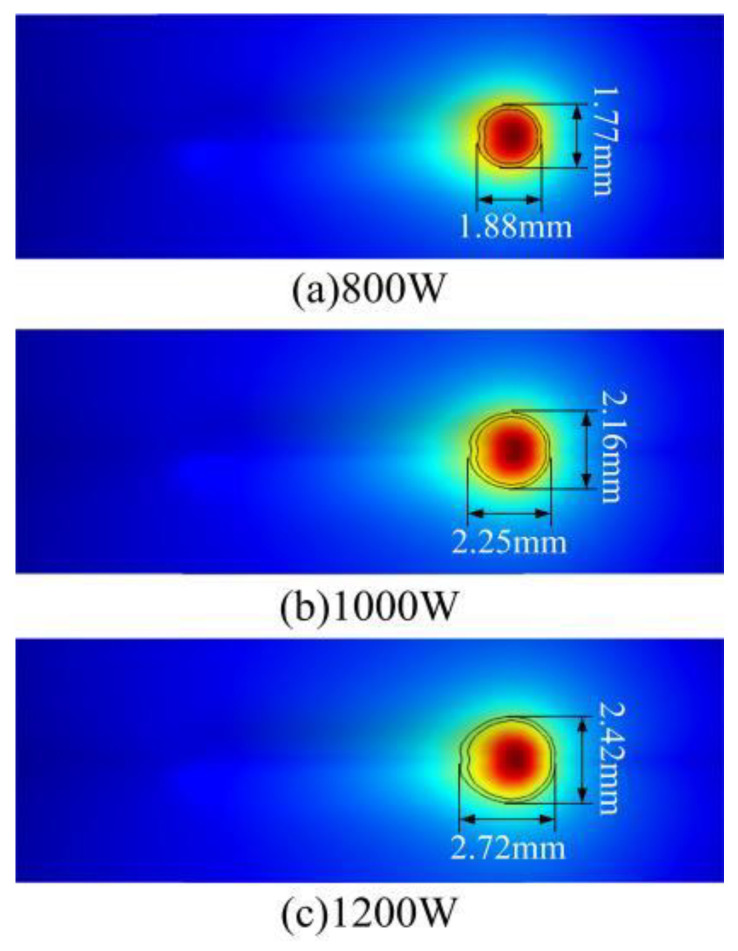
Simulation results of the morphology change of molten pool under different laser powers.

Under different laser power, the shape of the molten pool will change, which directly affects the cladding quality, process efficiency and the accuracy of the forming layer [37]. The morphological changes of molten pool under different laser power are shown in Figure 9. The two lines represent the solid phase line and the liquidus line respectively. As can be seen from Figure 9, with the increase in laser power, the width and length of the molten pool gradually increase. The high-power laser can input more heat, accelerate the melting rate of metal, and enhance the heat transfer effect. At the same time, the absorption rate of the laser energy of the rapidly heating substrate and powder increases, which makes the molten pool rapidly formed and expanded. In addition, with the increase of laser power, the peak temperature and flow rate of the molten pool surface gradually increase, resulting in greater wetting and fluidity inside the molten pool, which further enhances the evolution of the molten pool.

### 4.4. Analysis of Solidification Characteristics

**Figure 10:**
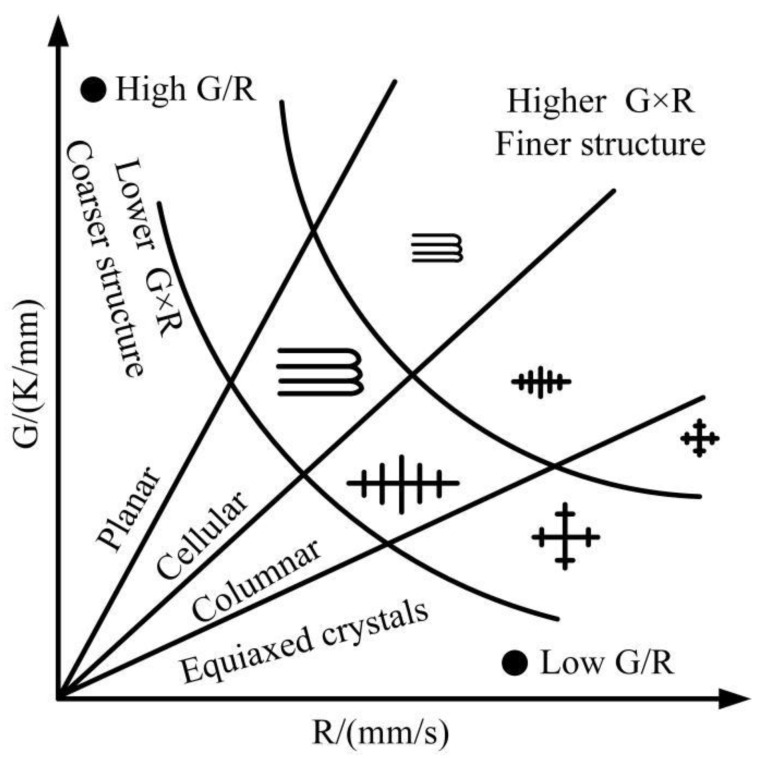
Effect of temperature gradient (G) and solidification rate (R) on the type and size of solidified tissue.

Temperature gradient (G) and solidification rate (R) are important drivers of alloy crystallization [38]. The numerical combination of temperature gradient and solidification rate (G × R, G/R) is an important parameter to describe the growth of dendrites, as shown in Figure 10. The cooling rate (G × R) has an important effect on the dimensions of the microstructure. The higher the cooling rate, the finer the microstructure size. The solidification parameters (G/R) affect the type and characteristics of the microstructure. As G/R decreases, the microstructure morphology changes from equiaxed crystals to cystalline, columnar, and equiaxed crystals.

**Figure 11:**
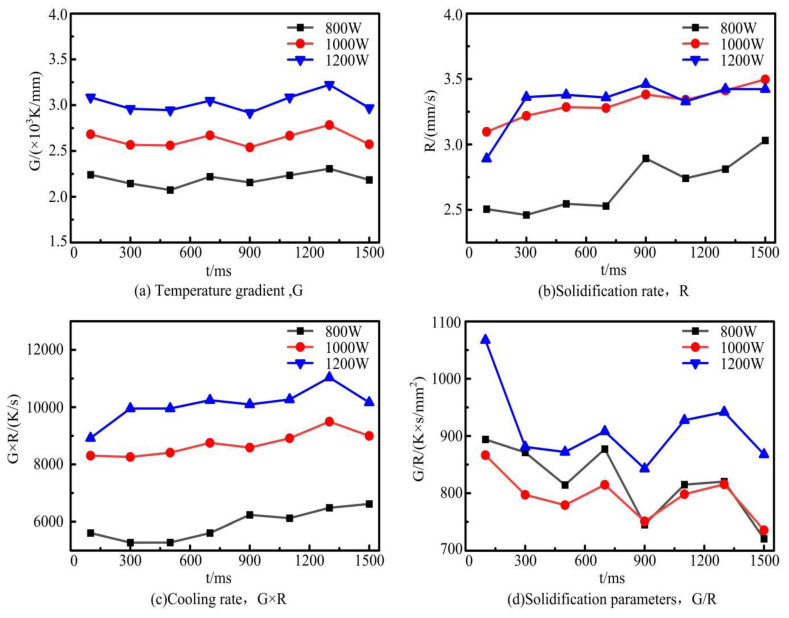
Simulation results of solidification characteristics of cladding layer under different laser powers.

In order to investigate the solidification characteristics of the melted cladding layer under different laser powers, eight probes were sequentially inserted in the X-axis direction to obtain the temperature gradient G and the solidification rate R. Among them, the solidification rate was obtained as follows. Five point domain probes are inserted in the direction of the depth of the melted cladding layer at the corresponding position of the X-axis probe, as shown in Figure 3d. Derive the temperature change curve at each point and then calculate the solidification rate in the depth direction of the cladding layer [39,40]. The solidification rate of the probes on the X-axis was selected for analysis. The variation rule of solidification characteristic parameters of the cladding layer with time under different laser powers is shown in Figure 11. As shown in Figure 11a, the temperature gradient fluctuates at different times. This is because the temperature rise of the material is not uniform when the laser is transmitted in the metal material. Due to the thermal convection and conduction effects, the temperature inside the melt pool fluctuates within a certain range, which causes the temperature gradient to fluctuate as well. The fluctuation trend of the temperature gradient of the cladding layer is approximately the same at different power levels. As the laser power increases, the energy absorbed by the molten pool in the same time increases, which leads to a faster heat accumulation. Therefore, the temperature gradient increases. As shown in Figure 11b, the solidification rate increases with the increase of laser power. The higher the laser power, the more intense the Marangoni flow inside the molten pool, and the more obvious the thermal convection and conduction effects, leading to a faster solidification rate. Because of the tendency for both the temperature gradient and the solidification rate to increase when the laser power increases, the product of the two increases. Therefore, the cooling rate (G × R) of the cladding layer is taken to be maximum at 1200 W. In contrast, the parameter (G/R) that determines the type and characteristics of the solidified tissue is taken to a minimum at a laser power of 1000 W. Based on the CET curves in Figure 10, it is predicted that the microstructure of the cladding layer is finer and denser at 1000 W.

## 5. Experimental Verification of Laser Cladding Incoloy 926 on 316L Surface

The experiment of laser cladding Incoloy 926 on 316L surface was performed using the same process parameters. By observing the macroscopic morphology, microstructure, and element distribution of the cladding layer, the reliability of the molten pool morphology, temperature field, solidification characteristics, and velocity flow field in the numerical simulation of laser cladding is verified.

### 5.1. Analysis of the Macroscopic Morphology of the Cladding Layer

**Figure 12:**
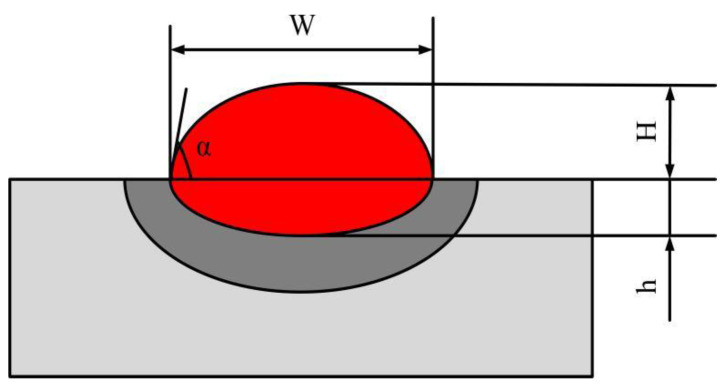
Schematic diagram of the macroscopic morphology of the cladding layer.

In the laser cladding process, the macroscopic morphology of the cladding layer directly determines the aspect ratio and surface roughness of the cladding layer, which in turn affects the molding quality [41]. The dilution rate (D) and wetting angle (α) of the molten cladding are the main parameters describing the macroscopic morphology. An excessive dilution rate will reduce the original properties of the substrate. A dilution rate that is too small will reduce the bonding strength of the fused cladding to the substrate [42]. If the wetting angle is too large, the cladding layer will be high and narrow and will be easily dislodged. Too small of a wetting angle will lead to excessive stress and crack initiation at the joint. The macroscopic morphology of the single-pass fusion cladding layer is schematically drawn as shown in Figure 12, and the dilution rate and wetting angle of the macroscopic morphology can be calculated by using Equations (12) and (13) [43]:

(13) $$D=\frac{h}{H+h}\times 100\%$$

(14) $$\alpha =2\arctan\frac{2H}{W}$$

where *W* is the width of the cladding, *H* is the height of the cladding, and *h* is the depth of the cladding.

**Figure 13:**
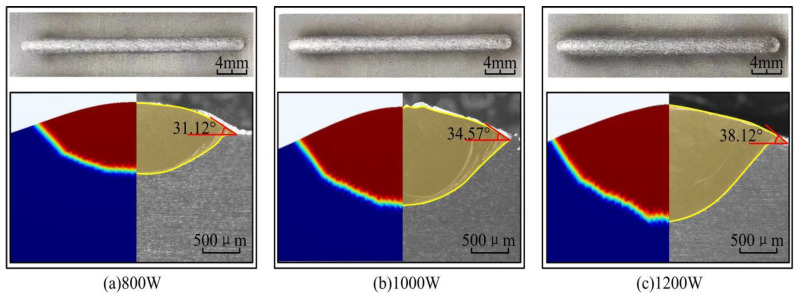
Macroscopic morphology of the cladding layer under different laser powers (simulation results on the left and experiment results on the right).

The macroscopic morphology of the laser cladding single-pass coatings on 316L surface prepared at different laser power is shown in Figure 13. The simulation results show the cross-sectional change of the molten pool in the phase field (0 is the solid phase, 1 is the liquid phase, and 0–1 is the mushy zone). When the laser power is small, the powder fails to fully melt due to the irradiation energy being insufficient. At the same time, the melt pool is not uniformly heated, resulting in unequal melting and solidification rates in different areas. These reasons lead to the uneven surface of the cladding. The cladding layer is high and narrow and very easy to flake off, resulting in poor quality of metallurgical fusion of the cladding layer. As the laser power increases to 1000 W, the surface of the cladding layer becomes relatively flat and shows metallic luster. This is because the high energy density can enhance the wetting and fluidity inside the molten pool, which promotes the uniform heat and mass transfer in the molten pool, thus effectively improving the molding quality of the cladding layer. However, when the laser power is too high, the substrate and the powder are overly liquefied due to the high temperature, resulting in darkening of the coating color. At the same time, the temperature gradient and solidification rate inside the melt pool increase, resulting in the powder adhering to the surface of the cladding layer before it is completely melted. This is also known as the phenomenon of sticky powder. The macro-morphological parameters of the cladding layers at different laser powers are shown in Table 3. As the laser power increases, the height, depth, width, dilution rate and wetting angle of the cladding layer all increase. The higher laser power inputs higher thermal energy into the molten pool, which promotes the widening and deepening of the molten pool. This is basically consistent with the results of numerical simulation. It is noteworthy that the depth of the molten pool increases dramatically when the laser power is 1200 W. This is mainly due to the high temperature of the metal solution caused by the evaporation phenomenon, which increases the absorption of laser energy in the center region of the melt pool and enhances the heat transfer. This phenomenon is prone to causing the keyhole effect, increasing the porosity of the cladding layer and affecting the molding quality and mechanical properties of the cladding layer [44]. At the same time, the consistently increasing dilution rate and wetting angle also illustrate that higher laser power is not always better.

### 5.2. Analysis of the Microstructure of the Cladding Layer

**Figure 14:**
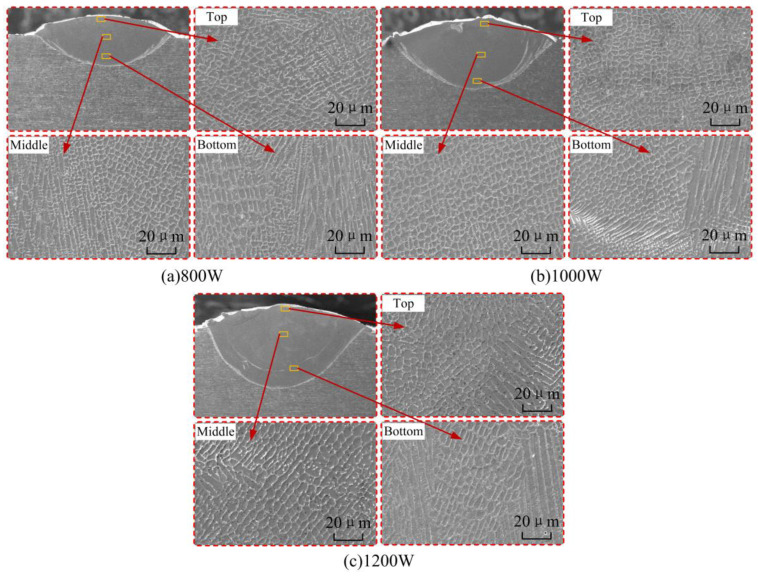
Experimental results of microstructure of cladding layer under different laser powers.

The microstructure of the cladding layer at different laser powers is shown in Figure 14. The forming quality of the cladding layer is good, with no pores and cracks in the microstructure. This is because the high-energy laser beam inputs high heat to the molten pool. Strong convection currents are generated while the melt pool temperature remains high for a short period of time, which helps to remove air bubbles from the melt pool. From the top to the bottom of the cladding layer, the microstructure changes from equiaxial diameter to columnar dendrites. When the laser power increases from 800 W to 1000 W, the cooling rate (G × R) higher larger and the solidification parameters (G/R) become lower. The microstructure of the cladding layer is refined and finer “honeycomb” isometric dendrites appear. When the laser power increases to 1200 W, the size of the dendrites increases again and the phenomenon of blurred grain boundaries appears. The experimental results are in general agreement with the analytical results of numerical simulations.

**Figure 15:**
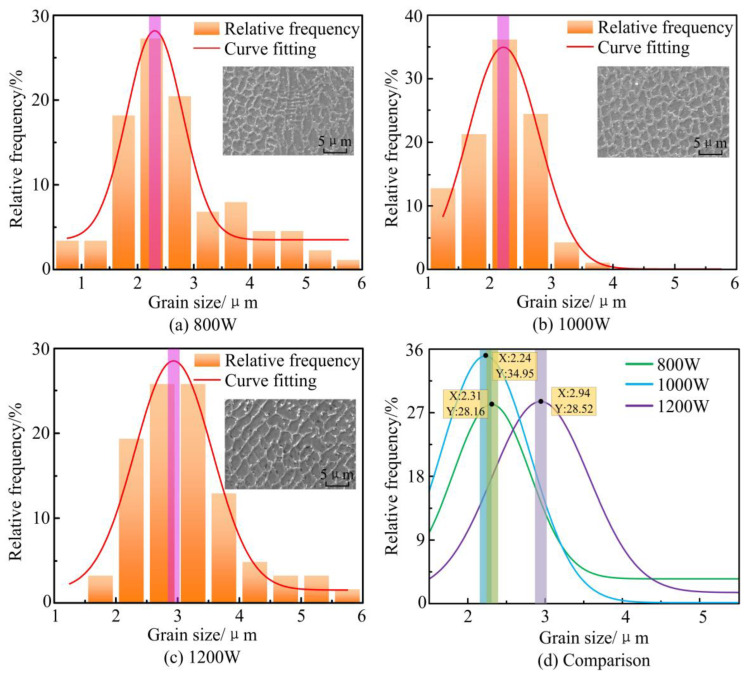
Experimental statistical results of grain size distribution in the middle of cladding layer under different laser powers.

In order to investigate the effect of laser power on grain size, the distribution of grain size in the middle of the cladding layer is statistically analyzed, as shown in Figure 15. The grain size distributions in the middle of the cladding layer at different laser powers are all approximately normally distributed, with average grain sizes of 2.31 μm, 2.24 μm, and 2.94 μm, respectively. The average grain size in the middle of the cladding layer is the smallest when the laser power is 1000 W.

### 5.3. Analysis of the Elemental Distribution of the Cladding Layer

**Figure 16:**
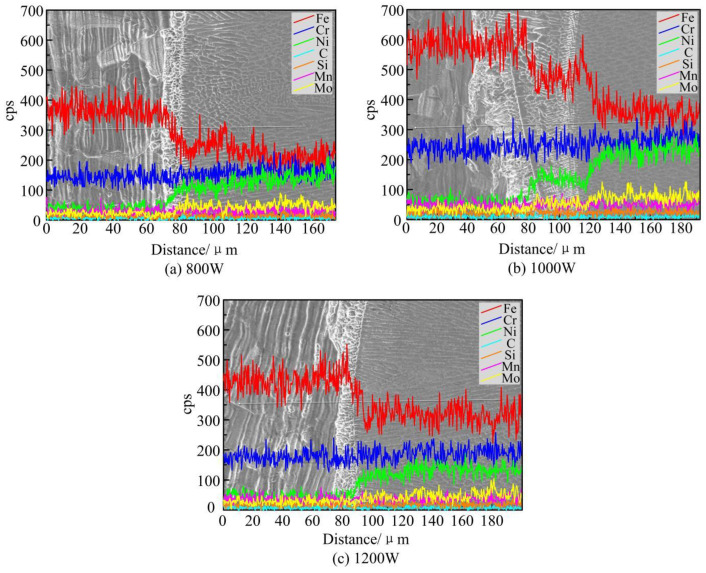
Experimental results of element distribution in cladding layer under different laser powers.

In order to investigate the effect of different power on the distribution of elements in the cladding layer, EDS line scanning was performed along the centerline of the cladding layer cross-section, as shown in Figure 16. The main elements in the cladding layer are Fe, Cr, Ni, C, Si, Mn, and Mo, which are the main chemical compositions of the substrate and powder. Among them, the element composition of Incoloy 926 alloy is mainly in the cladding layer region. In the heat-affected zone of the cladding layer, the content of the elements changed dramatically. Among them, the content of Fe element decreased and that of Ni element increased. This indicates that the alloying elements of the substrate and the powder produce a good mixing in the melt pool. This changes the relative proportions of the alloying elements in the cladding layer. As the laser power increases, the gradient of change of each element becomes relatively larger and the distribution is relatively more uniform. This is due to the fact that greater laser power intensifies the Marangoni flow in the region of the cladding layer. The elements between the powder and the substrate diffuse into each other, which promotes a good metallurgical bond between the cladding and the substrate. However, when the laser power is too high, the flow inside the molten pool is too violent. The diffusion of elements between the powder and the substrate becomes relatively worse and the degree of uniform distribution decreases.

## 6. Conclusions

In this paper, a three-dimensional thermal fluid multi-field coupling laser cladding numerical model is established by comprehensively considering the factors of convective heat transfer in the molten pool, transient dynamic evolution, solidification phase transition and so on. The influence laws of laser power on the temperature field, velocity flow field, molten pool morphology, and solidification characteristics of laser cladding Incoloy 926 on stainless steel surfaces are analyzed. The experiments of laser cladding Incoloy 926 on 316L surface were carried out under different laser power conditions. The influence of laser power on the geometrical morphology, microstructure and element distribution of the cladding layer was analyzed. By comparing the numerical simulation results with the experimental results, the following conclusions can be drawn:

(1) In the process of laser cladding, the molten pool is elliptically distributed and exhibits a symmetrical Marangoni flow inside. With the increase in laser power, the peak temperature and velocity of the molten pool surface increase gradually, which promotes the further expansion of the width and length of the molten pool. Both of them have the same trend in the axial direction. The thermal influence of the molten pool center on the edge increases gradually, especially the depth of the molten pool.
(2) When the laser power increases, the temperature gradient, solidification rate, and cooling rate of the molten pool gradually increase. While the microstructure parameters (G/R) are relatively small when the laser power is 1000 W.
(3) In the test range of laser cladding Incoloy 926 on 316L surface, with the increase in laser power, the dilution rate and wetting angle of the cladding layer both increase. When the laser power is 1000 W, the elements inside the cladding layer are more evenly distributed and the microstructure is finer. The experimental and simulated results agree well with each other.

## Figures and Tables

**Table 1:** Chemical composition of 316L stainless steel and Incoloy 926 alloy (wt.%).

	Fe	C	Si	Mn	P	S	Cr	Ni	Mo	N	Cu
316L stainless steel	68.7245	0.0179	0.6	1.372	0.0266	0.019	16.88	10.05	2.01	0.3	/
Incoloy 926 alloy	45.21	0.02	0.50	1.02	0.03	/	20.00	25.5	6.50	0.25	0.96

**Table 2:** Laser cladding process parameters.

Process Parameter	Symbol	Value	Unit
Laser Power	P_L_	800	W
		1000	W
		1200	W
Scanning Speed	v	6	mm/s
Powder Feeding Capacity	v_p_	1.6	r/min
Defocusing amount	d	16	mm
Spot Radius	R_L_	1	mm
Melting Radius	r	1.5	mm
Effective Cladding Depth	h	1	mm

**Table 3:** Macro-morphological parameters of the cladding layer at different laser powers.

	H/μm	h/μm	W/μm	D (%)	Θ (°)
800 W	265.80	415.08	1909.12	60.96	31.12
1000 W	374.57	604.49	2407.41	61.74	34.57
1200 W	429.84	723.53	2488.46	62.73	38.12

## Data Availability

The original contributions presented in the study are included in the article, further inquiries can be directed to the corresponding author.
